# Mucins as Precision Biomarkers in Glioma: Emerging Evidence for Their Potential in Biospecimen Analysis and Outcome Prediction

**DOI:** 10.3390/biomedicines12122806

**Published:** 2024-12-11

**Authors:** Anna Erickson, Luke R. Jackson, Kevin Camphausen, Andra V. Krauze

**Affiliations:** Radiation Oncology Branch, National Cancer Institute, National Institutes of Health, Bethesda, MD 20892, USA; anna.erickson@nih.gov (A.E.); luke.jackson@nih.gov (L.R.J.); camphauk@mail.nih.gov (K.C.)

**Keywords:** MUC, mucins, omics, glioblastoma, chemo/radioresistance, recurrence

## Abstract

Despite attempts at improving survival by employing novel therapies, progression in glioma is nearly universal. Precision biomarkers are critical to advancing outcomes; however, biomarkers for glioma are currently unknown. Most data on which the field can draw for biomarker identification comprise tissue-based analysis requiring the biospecimen to be removed from the tumor. Non-invasive specimen-based precision biomarkers are needed. Mucins are captured in tissue and blood and are increasingly studied in cancer, with several studies exploring their role as biomarkers to detect disease and monitor disease progression. CA125, also known as MUC16, is implemented as a biomarker in the clinic for ovarian cancer. Similarly, several mucins are membrane-bound, facilitating downstream signaling associated with tumor resistance and hallmarks of cancer. Evidence supports mucin expression in glioma cells with relationships to tumor detection, progression, resistance, and patient outcomes. The differential expression of mucins across tissues and organs could also provide a means of attributing signals measured in serum or plasma. In this review, we compiled existing research on mucins as candidate precision biomarkers in glioma, focusing on promising mucins in relationship to glioma and leading to a framework for mucin analysis in biospecimens as well as avenues for validation as data evolve.

## 1. Introduction

Gliomas represent the most common malignant primary brain tumor [1]. In 2021, the World Health Organization published their fifth edition of the Classification of Tumors of the Central Nervous System, with adult and pediatric gliomas classified separately based on differences in molecular pathogenesis and prognosis [2]. The adult-type diffuse gliomas were consolidated into three types: astrocytoma, isocitrate dehydrogenase (IDH) mutant; oligodendroglioma, IDH mutant and 1p/19q co-deleted; and glioblastoma, IDH wildtype [3]. These changes were driven by IDH mutation status, leading to glioblastoma being considered grade IV [4]. Given their infiltrative and treatment-resistant behavior, gliomas are associated with poor prognosis, especially in the context of glioblastoma (GBM), with a median survival of less than 2 years and a 5-year survival rate of less than 6% [5,6]. Although the current standard of care comprises maximal tumor resection followed by chemotherapy and radiation therapy, patients with glioma experience nearly universal eventual recurrence, and the disease remains incurable [7,8]. Several therapeutic avenues have been explored to improve outcomes in both low- and high-grade glioma by augmenting the standard of care, including immunotherapy, and nanontherapies including attempts at linking molecular classification to response via key diagnostic genes [9,10]; however, the malignant nature of gliomas is multifactorial and grounded in significant intra- and inter-tumor heterogeneity [7]. The observed heterogeneity is, in part, related to the origin of gliomas as they arise from neuroglial stem/progenitor-like cells, leading to both genetic and epigenetic malignant modifications [11]. Tumor heterogeneity is considered one of the most impactful contributors to tumor recurrence and treatment failure [12]. Tumor behavior and resistance have also notably been linked to the ability of tumor cells to reconfigure the tumor microenvironment by employing various signaling pathways that alter cell adhesion and facilitate immune evasion by directing surrounding cells. These mechanisms eventually lead to tumor resistance and progression [8].

Mucins are a family of highly glycosylated proteins ubiquitously expressed, whose evolving roles include modulation of the tumor microenvironment and growing relationships to tumor resistance and proliferation. Several mucins have been reported in GBM, including MUC1, MUC4, and MUC16 [6,7,8], as well as several others. Mucins are highly measurable in the blood and have wide-ranging roles [13] and transferable use in several diseases [14]. Several mucins have shown clinical promise, including MUC1 (also known as CA 15-3 and KL-6, in breast cancer [13,15]), MUC4 (pancreatic cancer [16]), and MUC5 (gastrointestinal, pancreatic, gastric cancers [13,17,18]); however, only one is FDA-approved and in clinical use, MUC16, also known as CA125 in ovarian cancer [19,20]. Given MUC16 as a precedent for the clinical implementation of a mucin biomarker and growing evidence for mucin detection and association with outcomes in glioma, a careful evaluation of mucins as potential non-invasive precision biomarkers in GBM is indicated to help direct and interpret findings in this space. This review aims to (1) explore the biological role of mucins and their applicability to glioma, (2) evaluate means of measurement and levels of mucin detection, and (3) provide an exploratory framework for the study and advancement of mucins as precision biomarkers in the glioma setting.

## 2. Biological Role of Mucins and Their Role in Glioma

**Figure 1:**
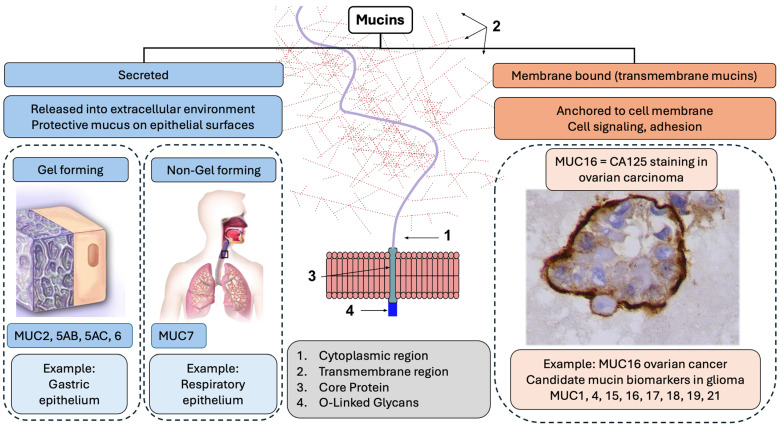
Mucin classification, organ specificity, and potential candidate mucins as biomarkers in glioma. The blue left panel illustrates secreted mucins, both gel-forming and non-gel-forming, identified in heavily glycosylated layers such as the gastric epithelium and non-gel forming as identified in the respiratory epithelium. The right panel illustrates membrane-bound mucins, including MUC16, currently in use in ovarian cancer, as well as candidate mucins in glioma (MUC1, MUC4) [31,32,33,34,35].

Mucins are a family of highly glycosylated proteins with one region of the polypeptide backbone rich in threonine and serine residues as well as O-glycosylated domains containing proline that are covalently bound and detected on the surface of cells, both benign and malignant [20] (Figure 1). These three amino acids are known as the proline-threonine-serine (PTS) domain, and their tandem-repeat nature is common to all mucin fields [21]. PTS domains are responsible for the biochemical and biophysical properties of mucus because of their essential role in glycosylation [21], and, as such, they are potentially highly transferrable biomarker candidates. They also represent the most abundant macromolecules in mucus, which provide a protective barrier throughout the body against harmful substances [22], playing an essential role in the renewal and differentiation of the epithelium. They also have a critical role in cell adhesion, immune response, and cell-to-cell signaling [23]. Increased synthesis of mucins and alterations made to carbohydrates attached to mucins are implicated in the proliferation of tumor cells with documented overexpression in several cancers [24]. At least 20 genes have been identified in *Homo sapiens* that give rise to gel or secreted mucins [25]. These genes are designated as MUC1 to MUC 22. They are classified into secreted and membrane-bound mucins according to their structure and location, with the number representing the sequence of their discovery [25] (Figure 1). There are two sub-classes of secreted mucins: gel-forming (MUC2, MUC5AC, MUC5B, MUC6) and non-gel-forming (MUC7) [26,27] (Figure 1). Their highly repetitive structure recognizes gel mucins and the von Willebrand D (VWD) domain [28]. The three-dimensional structure of the VWD domain needs to be better understood, but evidence points to its involvement in the polymerization of mucin monomers through intermolecular disulfide bonds [26]. Genes that encode gel-forming mucins have some similarities and are believed to have arisen via the duplication of a common ancestor [29]. Secreted mucins are unique in that they show patterns of expression restricted to secretory organs and cell types [19]. They provide a protective layer over the organs, working as a barrier against pathogens [20]. In addition to aiding in protection, they have a role in signaling, monitoring, and repairing damaged epithelia [30]. MUC2 represents one such example and has been found to play a role in suppressing inflammation in the intestinal tract, which has been linked to its anti-tumor ability within the intestines [28].

Membrane-associated mucins are bound to cells by an integral transmembrane domain and contain short cytoplasmic tails with adaptor proteins that participate in signal transduction [36] (Figure 1). Many of these mucins have juxta-membrane domains with homology to the epidermal-growth-factory (EGF) family [37].

MUC1 specifically has been shown to interact with STAT3 and other growth factors and cytokines including TNFα. This signaling connection is extremely relevant because STAT3 is implicated in several cancers including glioma [38]. In addition, MUC1 is also linked to the PI3K/AKT pathway and VEGF secretion through both HIF-1α-dependent and -independent mechanisms [39]. This aspect links mucins to angiogenesis, which is particularly relevant to glioma [40].

**Figure 2:**
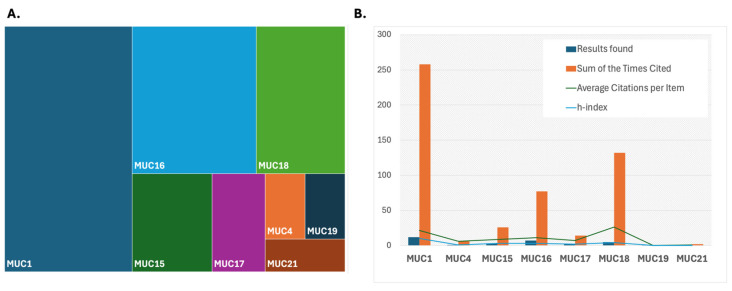
(**A**). Tree diagram illustrating the presence of mucins in the literature based on Web of Science search [42] for each numbered mucin as an individual term and the presence of the term “glioma” in the same article. Several mucins did not have a literature presence in glioma and therefore, do not feature in the tree diagram. (**B**). Illustration of the mucins identified in connection with glioma based on data from Web of Science with average citations per item, sum of times cited and h-index as proxies for interest delegated in the field towards specific mucins.

While the mechanisms are not fully understood, there are growing data that these domains interact with EGF receptors, which have downstream impacts on regulation or signaling related to growth, motility, differentiation, and inflammation [39,41] (Figure 2). To better understand the impact of mucins in this space, a systematic search was conducted in the Web of Science (WoS) database to analyze publication trends and citation metrics for mucins (proteins classified under the mucin family). The search spanned the full temporal range available in WoS, from 1900 to 2024. Individual mucins were identified by their specific names or abbreviations (e.g., MUC1, MUC2, etc.), corresponding to their numerical designation based on the order of discovery. Separate searches were performed for each mucin, retrieving publication records for titles, abstracts, and keywords containing the molecule’s name. For each mucin, the following bibliometric data were extracted: the total number of publications, the sum of times cited, the average citations per publication, and the h-index. These data (Supplemental Table S1) were then exported from WoS and compiled into a dataset for analysis. The results were visualized using two main approaches employing Excel V. 2409: (1) a tree map diagram to illustrate the relative contributions of individual mucins to the total publication and citation landscape and (2) a bar graph to display the bibliometric indicators for each mucin (number of publications, total citations, average citations per item, and h-index). These visualizations were generated to identify trends and highlight differences in research focus and impact across the mucin family. Membrane-bound mucins are, as a result, biologically and clinically applicable in glioma. Evidence for using mucins as relevant molecules in glioma has been growing.

The physiological role mucins play in the formation of protective barriers is amplified in neoplastic transformation. In general, mucins are highly involved in forming the extracellular matrix and, thus, contribute to forming the tumor microenvironment in most solid tumors. Alterations in the expression, distribution, and glycosylation of membrane-associated mucins have been linked to the invasive and metastatic properties of adenocarcinomas by altering the adhesive properties of the surface of the tumor [19]. Additionally, this barrier can lead to chemotherapy resistance by limiting drug penetrance into the tumor, masking surface antigens, and triggering epithelial-to-mesenchymal transition, which is a significant step toward metastatic progression and drug resistance [43]. Additionally, mucins modulate inflammation and immune responses in extracranial cancers by directly interacting with immune cells and cytokines [44]. While glioma is not metastatic beyond the CNS, epithelial-to-mesenchymal transition (leading to chemotherapy and radiation resistance) and immunosuppression are both highly pathogenic characteristics of glioma [45,46]. Mucin involvement in extracranial tumor cell processes merits investigation in glioma, especially since preliminary studies in glioma have identified mucins as drivers in pathways important to glioma progression and resistance [47,48,49,50].

Mucins are heavily interactive with each other, with strong confidence based on several sources of interaction. To better illustrate this, a protein–protein interaction (PPI) network was constructed using the STRING database (Search Tool for the Retrieval of Interacting Genes/Proteins) (version 11-5.string-db.org) to explore interactions between mucins and the glioma molecular markers IDH1 and MGMT with query parameters, including the mucins in Figure 1, IDH1, MGMT, and EGFR. IDH1 was selected as this is the primary IDH molecule employed for molecular classification in glioma. STRING was accessed to retrieve data on known interactions, predicted interactions, and other interaction types, using the full STRING network to provide a comprehensive analysis. All interactions involving IDH1 and MGMT were included to highlight connections relevant to glioma biology. The edges in the resulting network were annotated with evidence supporting the interactions, including experimental data, co-expression patterns, text mining results, gene co-occurrence, and database annotations. These evidence types were used to classify interactions as known, predicted, or others based on STRING’s scoring system (Supplemental Table S2). The network visualization was generated to represent proteins as nodes and interactions as edges, with edge thickness reflecting the confidence score of the evidence. The interactions displayed were then limited to mucins identified in glioma (Figure 2) to allow for visibility of the edges and interactions. This analysis aimed to elucidate the molecular interactions of mucins with IDH1 and MGMT via EGFR, providing insights into their potential roles in glioma-related pathways and broader biological processes, and the resulting PPI enrichment *p*-value was <1.0 × 10^−16^, ranging from 0.99 to 0.42 (Supplemental Table S2).

**Figure 3:**
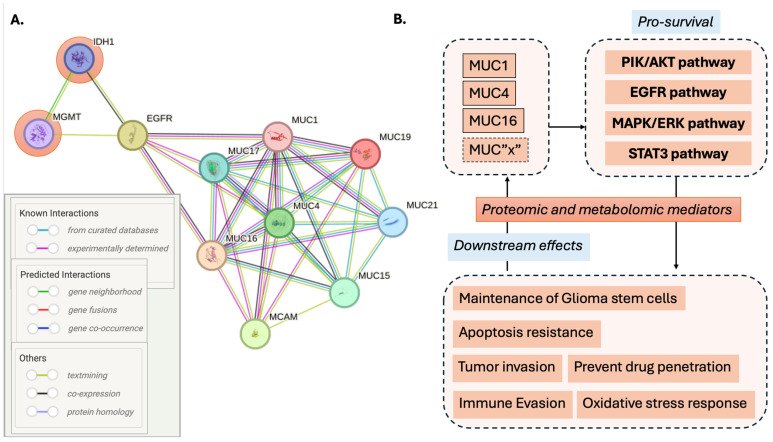
(**A**). STRING diagram showcasing interactions of mucins identified in glioma with solid connections to EGFR and potential connections to IDH1, and MGMT as known glioma molecular markers [72]. (**B**). Promising mucins in glioma illustrating connections to pro-survival pathways and downstream effects. Precision markers may be identified amongst candidate mucins and proteomic and metabolomic mediators connected to molecular classification.

According to the visualization of their interconnected nature in STRING, as illustrated in the context of several mucins identified as potential precision biomarkers in GBM (Figure 3A), evolving evidence for this interaction has implicated mucins as actors in pro-survival pathways (Figure 3B), including via EGF families and the PIK/AKT and MAPK/ERK pathways. Downstream effects of these interactions include maintenance of the stem cell population, resistance to apoptosis, immune evasion, and oxidative stress response. Several of these mechanisms are of significance to treatment resistance in glioma. In glioma, EGFR signaling supports the connection of mucins to molecular classification (Figure 3A). MUC4 was found to be co-expressed with MMP9 and EGFR GBM tissue [51], and a combined high expression of MUC4/MMP9 and MUC4/MMP9/EGFR was associated with adverse outcomes [49,52]. Based on CRISPR-Cas9 screening, it was found that MUC1 is essential for EGFRvIII glioma cell survival and TMZ resistance as it was upregulated in EGFRvIII-positive cells [53]. The MUC1 gene encodes a single polypeptide chain that is cleaved into a longer N-terminal subunit (MUC1-N) and shorter C-terminal subunit (MUC1-C) because of conformational stress [54]. While mucins have been detected in various cancers, their role in glioma is evolving, given their well-known association with gel-forming layers and non-gel-forming protective aspects in organ settings outside the brain, where the study of mucins has progressed more rapidly. More recently, it has become clear that membrane-bound mucins associated with signal transduction can have significant implications in the context of glioma. Specific mucins that have now been implicated in glioma include MUC1, MUC4, MUC15, MUC16, MUC17, MUC18 and MUC21 (Figure 2, Table 1) [50,55]. Similarly to MUC16 in ovarian cancer, these mucins are all part of the membrane-bound mucin family, which possesses a more significant number of O-glycosylation sites and more tandem repeats [56], the former being a shared feature of secreted and membrane-bound mucins (Figure 1). Data collected for MUC16 mRNA expression in 37 cancer types originating from the Cancer Genome Atlas were analyzed, and MUC16 was frequently found in low-grade glioma (LGG), leading to further research that indicated mutated MUC16 in LGG was associated with a better prognosis overall [24], while in GBM patients, the same mutation resulted in a worse prognosis [24]. Protein expression in patients with grade II-IV gliomas demonstrated that MUC4 is significantly upregulated during glioma progression, leading to the potential for a role in proliferation and angiogenesis [49].

MUC15 has also benefited from exploration in glioma (Figure 2) and was found to mediate signal transduction [66]. Work conducted by Cheng and Liu led to the finding that MUC15 activates the Raf/MEK/ERK signaling pathway, and specific ERK inhibitors reversed MUC-15’s enhanced proliferation and invasion of glioma cells, which indicates that MUC15 may have a significant role in glioma tumorigenesis [60]. MUC17 has also been reported on and, although not explicitly identified in the Human Protein Atlas as a candidate based on single-cell fractional expression data, it was nonetheless found associated with poor prognosis in both non-GBM and GBM cohorts [55,57,67]. Meanwhile, MUC18, also known as CD146 and MCAM, has benefitted from significant literature interest (Figure 2), as it has been associated with glioma based on publications that have been in the public domain for a longer period. It was reported in conjunction with the loss of AP-2alpha as one of several genes with AP-2alpha binding motifs that also included E-cadherin, p21WAF1, MMP-2, VEGF, and c-KIT [68]. It was found to have increased expression in dividing glioma stem cells [61] and has been researched extensively in melanoma, given its upregulation in tumors of neuroectodermal origin. MUC19 and MUC 21 are actively evolving in glioma. MUC19 was identified in a recent proteogenomic analysis in GBM [62]. MUC21 is expressed as a large glycoprotein at the cell surface and an inhibitor of cell–cell and cell–matrix adhesion. GBM tissues display upregulation, and MUC21 promotes GBM cell viability and migration in vitro [50,69].

In non-glioma settings, MUC1-C has been associated with metabolic reprogramming in esophageal squamous cell carcinoma (ESCC) via the inhibition of the AKT pathway, making MUC-1 a potential target for treating ESCC [70]. Meanwhile, MUC4 overexpression in mammary epithelial cells significantly inhibits ERK1/2 phosphorylation, an interaction required to activate the ERK-MAPK pathway [71].

## 3. Means of Mucin Measurement and Levels of Detection in Glioma

Previous data have shown promise for using mucins as biomarkers captured in the blood in several malignancies [73,74] (Table 1). Mucins have been linked to carcinomas of the pancreas, colon, lung, ovary, and breast [16,75,76,77]. In the case of colon cancer, the loss of MUC2 and MUC4 and the gain of MUC4, MUC5AC, and MUC16 are associated with oncogenic progression [78,79,80]. There has been extensive study of mucins in various cancers as a means of cancer detection and monitoring [13]. The most prominent examples are CA125, also known as MUC16 for ovarian cancer, CA19-9 for pancreatic cancer, and KL-6 (also known as MUC1) for breast and lung cancer. MUC1 is overexpressed in pancreatic, lung, breast, colon, and ovarian cancers, while MUC4 overexpression has been observed in colon adenocarcinoma and pancreatic cancer [43,65]. In addition, mucins have been detected in serum for gastric cancer diagnosis. Studies have shown that MUC5AC apomucin originating from cholangiocarcinoma (CCA) tissues was detected in patient serum with high sensitivity and specificity [18]. Further analysis of MUC5AC found in serum revealed an association with survival rates in patients with CAA [17]. Meanwhile, MUC16 has been extensively researched as a biomarker in ovarian, pancreatic, and bladder cancer, with the clinical application of CA125 in ovarian cancer [81], where MUC16 is FDA-approved as a biomarker for monitoring ovarian cancer recurrence [82].

**Figure 4:**
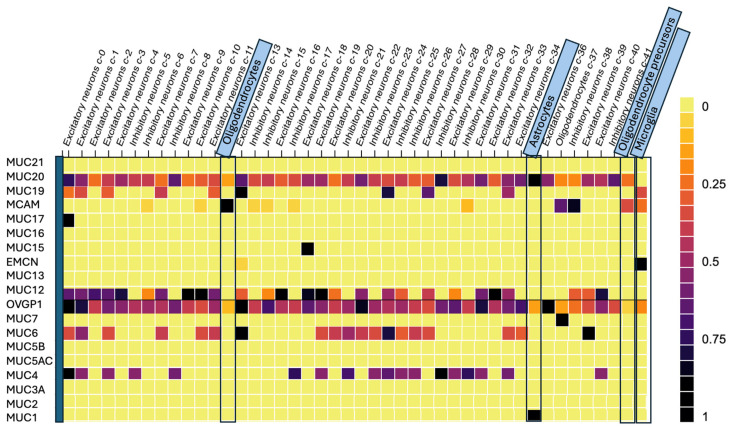
Heat map representing expression levels of mucins in various glial cells as identified and adapted from the Human Proteome Atlas [83]. The vertical dashed lines highlight glioma cells of origin (oligodendrocytes, astrocytes, oligodendrocyte precursors and microglial cells). Mucins (left vertical arrangement top to bottom from highest to lowest assigned number i.e., order of discovery) are displayed based on the fraction of highest expression in various cells of neural origin. OVGP1, EMCN and MCAM have alternate names in the mucin family: OVGP1 (MUC9), EMCN (MUC14) and MCAM (MUC18). MUC1 is exclusively expressed in astrocytes. MCAM (MUC18) is nearly exclusively expressed in oligodendrocytes, oligodendrocyte precursors and to a lesser extent microglial cells.

Several mucins are of interest; however, expression in neural tissues is critical. To examine this aspect, the expression levels of mucins in various glial cell types were analyzed using data obtained from the Human Protein Atlas [83]. The analysis focused on glial cells of neural origin, including glioma cells of origin: oligodendrocytes, astrocytes, oligodendrocyte precursors, and microglial cells. Expression data for each mucin were exported and visualized in descending order of discovery (highest to lowest assigned mucin number). To enhance clarity, a heat map was generated by taking the relative expression levels of each mucin across the cell types. The fraction of the highest expression value was used to standardize comparisons in the Human Protein Atlas, and this scale was employed to compare mucin expression (Figure 4). Vertical dashed lines were included to highlight specific glioma cells of origin. Special attention was given to mucins with alternate names in the mucin family, namely OVGP1 (MUC9), EMCN (MUC14), and MCAM (MUC18), with their dual nomenclature indicated for reference.

MUC1 showed exclusive expression in astrocytes, while MCAM (MUC18) demonstrated predominant expression in oligodendrocytes, oligodendrocyte precursors, and microglial cells. These findings provide insights into the cellular localization of mucins in neural tissues.

MUC9, MUC12, and MUC20 exhibit broad expression across neuronal cells but have differential expression between astrocytes and oligodendrocytes (Figure 4). While MUC1, MUC4, MUC15, MUC16, MUC17, and MUC21 are all potential candidate biomarkers for glioma, the extensive research on MUC1 and the current clinical application of MUC16 in ovarian cancer highlights their promise as therapeutics for glioma. MUC1 and MUC16 are also the two mucins directly connected to EGFR with co-expression and experimental data (Figure 3A). MUC4 appears to be a central player in mucin interaction, with experimental data connecting it to nearly every other mucin, despite no direct specificity for glioma. Given the extensive study of mucins in various malignancies to detect [13], monitor, and develop novel therapy [84], along with their distinct and potentially tissue-specific expression, mucins represent practical and promising biomarkers. Their evolving linkages to cancer outcomes enforce the growing understanding of some mucins as disease-directing entities. It should be noted that MUC1 and MUC16 are the only two mucins that, according to the Human Protein Atlas [83], can be measured in blood, making them the most applicable for therapeutics. MUC16 has also been expressed in several brain cell lines and has continued to be standard practice for ovarian malignancies, given its ability to be measured in blood. Because of this, we see a potential impact from MUC16 in malignancies beyond ovarian cancer. While there is a paucity of studies that analyze mucin levels in serum, tissue, and CSF samples in glioma patients, there are malignancy settings that have employed mucins as biomarkers in several or all of these biofluids, indicating that such an approach is feasible (Table 2).

## 4. Mucins as Biomarker Candidates in Glioma—A Framework for Analysis and Evaluation

**Figure 5:**
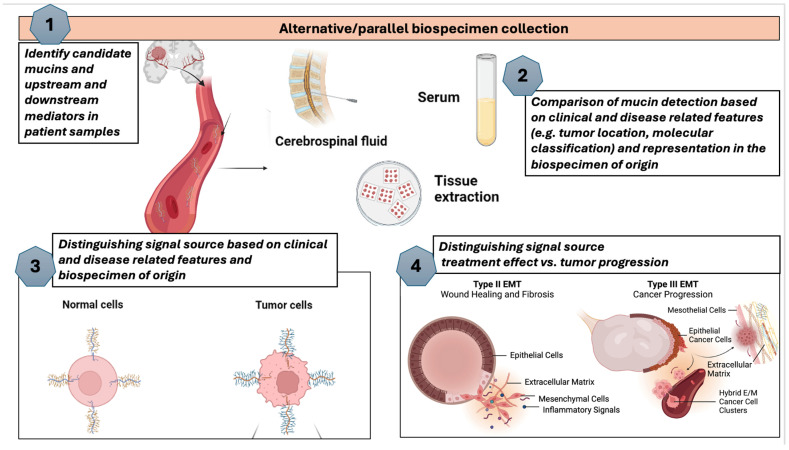
Proposed framework for mucin analysis as precision biomarkers in glioma illustrating potential steps that may be carried out concurrently or sequentially. (**1**). Identification of promising mucin molecules and the most promising upstream and downstream mediators or associated molecules based on existing data. (**2**). Characterization of mucins based on clinical and disease-related features such as tumor location and, specifically, biospecimen of origin. (**3**). Analyses aimed at distinguishing mucins as attributable to signal sources belonging to normal or tumor cells and biospecimen of origin. (**4**). Analyses aimed at distinguishing signal sources related to treatment effect comprised of wound healing postoperatively and fibrosis post chemo irradiation in glioma vs. tumor progression distinguished mucin signatures associated with active cancer cells [34]. The illustrations in this figure were created with the help of BioRender [99].

The current gold standard for GBM diagnosis is tissue obtained during tumor resection [91,92]. Still, this sample acquisition does not allow for further monitoring and is subject to sampling bias since only tissue safe for resection is sampled. Repeated tumor sampling is not clinically acceptable outside of tumor progression. It is only possible in this context if tumor location and clinical factors, including performance status, permit a re-resection [93]. Mucins can be captured in both tumor tissue and biospecimens, including blood and cerebrospinal fluid, with expression levels being highest in serum in some malignancies, as evidenced, for example, in breast cancer [94]. However, the signal captured in serum requires validation through comparisons with other biospecimens, the ability to distinguish its origin between normal cells and tumor cells, and the attribution of measured values as belonging to a treatment effect or tumor progression (Figure 5).

The proposed framework (Figure 5) involves identifying mucins and directly associated or mechanistically connected molecules (Step 1) followed by a comparison with the same signals in alternate biospecimens (Step 2). Distinguishing the source of the signal (Step 3) will require assigning signal specificity to the cell type using experimental assays and tissue correlation before moving on to distinguishing the treatment effect from tumor progression in Step 4. It should be noted that the half-life of MUC1, for example, in the plasma membrane, has been estimated at 16–24 h, with each round of sialylation, the modification of proteins by the addition of a sialic acid unit to the end of an oligosaccharide chain, taking around 2.5 h [95], suggesting that MUC1 could undergo significant recycling, impacting the measurement [96,97]. This can render the measurement of mucins variable. It is difficult to extrapolate the actual rate of recycling and cell-surface half-life in vivo due to varying recycling rates between cell lines and environmental conditions playing a crucial role [98]. However, these high recycling rates could be the key to understanding alterations in values between various experimental conditions, and, thus, they need to be controlled rigorously.

Plasma as a means of treatment monitoring in glioma has been subject to extensive research, emphasizing biomarkers, such as circulating tumor cells (CTCs), circulating tumor DNA (ctDNA), microRNA, and extracellular vesicles (Figure 5) [47,67,100,101]. The measurement of mucins when employing plasma sampling requires further research. Cerebrospinal fluid (CSF) provides a unique opportunity for biospecimen study in glioma, and detecting mucins in cancer has been made possible via CSF flow cytometry. The overexpression of MUC-1 was identified as a cell marker in breast cancer metastasis to the central nervous system using CSF flow cytometry, wherein 13 cases of breast cancer with leptomeningeal metastasis were successfully characterized as breast cancer leptomeningeal metastasis in all samples, despite a low cell count [63]. However, CSF as a means of mucin measurement was less successful in central nervous system lymphoma [102]. Currently, limited studies have been published evaluating the efficacy of CSF in detecting mucins in GBM. In CSF sensitivity and specificity for GBM detection were 80% and 67%, respectively, but when derived via lumbar puncture, sensitivity and specificity were detected at 28% and 95% [103]. These significant discrepancies highlight the inconsistency of CSF and the need to repeat studies on a larger scale. This study also expressed the challenges for obtaining these samples as they can only originate from patients with an Ommaya reservoir, a small dome-shaped device placed under the scalp [104], or a ventriculoperitoneal shunt. This makes CSF testing less accessible, limiting large-scale patient data.

Studying mucins and how they should be monitored in the context of GBM is critical to advancing therapeutics. An important avenue for defining the role of mucins is to examine their expression and interaction with molecules that are co-expressed throughout the analysis framework, from discovery to biological and clinical validation in clinical settings (Table 3). Interestingly, mucins can withstand high temperatures and different forms of radiation. Lieleg et al. performed a variety of treatments on both lyophilized and solubilized mucin samples, including thermal treatments, autoclaving, UV irradiation, and γ-rays. For all mucin samples, mucins were found to have excellent lubricity and friction curves that were nearly identical to untreated mucins. It was proposed that the high glycosylation density of mucins and lack of folding are a reason for the sturdiness of mucins toward heat shock [105]. Although mucins did have a significant loss of function when autoclaved and treated with γ-irradiation or UV light, they still provided excellent lubricity, independent of treatment time in the lyophilized state compared to the soluble state [106]. These results show the potential role mucins may have in resistance to chemotherapy and radiotherapy while also highlighting their importance as measurable signals across platforms and as therapeutic targets.

## 5. Measurement of Mucins in Metastatic Settings, Including the CNS

Inflammation upregulates mucin expression, which has been shown in several cancer and non-cancer conditions. However, mucin expression, in turn, can itself increase inflammation, alter cellular behavior, and, especially, transmembrane mucins can result in reprogramming towards epithelial-to-mesenchymal transformation and implicitly eventual metastasis [114]. Mucins can also circulate, evading immune system surveillance. This aspect connecting mucin secretion to metastasis has led to connections between cancer stage and detectable mucin levels in serum, including for CA-125 in ovarian cancer. It is not clear why higher levels are measured and whether they relate to tumor burden directly or as byproducts of mechanisms that allow cancer to spread. It is intriguing that malignancies with a propensity for metastasis to the brain have been associated with the secretion of certain mucins. This includes breast [115] and lung [116] cancer. In breast cancer, MUC1 was connected to resistance to Her2 targeting therapy with MUC1-mediated glycoprotein signatures identified in brain metastases [115]. Given its expression on the surface of cells in several cancers, it has been exploited for nanoparticle delivery in conjunction with fractionated radiation therapy employing MUC1-conjugated nanoparticles in breast and lung cancer [117]. In lung adenocarcinoma, MUC5AC was found enriched in brain metastases, with cells expressing MUC5AC progressing to brain metastases via annexin A2 as well as interplay with MMPs [116]. A similar mechanism may perpetuate the propensity for metastasis to the brain in breast cancer via signaling pathways, including MAPK, NF-κB, and STAT3 signaling in macrophages, which results in the production of the chemokines IL-6 and TNFα [118]. Mucin accumulation in brain lesions has been described in both primary brain tumors, where it has been associated with IDH mutation, and in brain metastases [117]. From a broader perspective, the role of mucin glycosylation in the gut may affect brain function and brain protein expression, including GFAP, NCAM, and Ki67, potentially also affecting the blood–brain barrier [119].

## 6. Conclusions

The ongoing lack of improvement in glioma outcomes renders the identification of precision biomarkers critical. Mucins can offer promising avenues as glioma biomarkers as they can be measured non-invasively in serum and plasma. Based on the current literature, expression profiles, and biological properties, MUC1, MUC4, MUC16, and MUC18 are of interest as precision biomarkers in glioma. However, other mucins may also prove highly critical depending on their expression profile in the tumor state, as compared to physiologic function. Their study in glioma is actively evolving, as is the ability to determine how robustly they may be measured in plasma or serum. Increasing data demonstrate that mucins can be linked to glioma grading and outcomes with differential expression between glioma subtypes. Their connection to EGFR supports their potential role in directing signaling and merits additional study. Evolving evidence and ongoing studies based on sampling biofluids can elevate the role of mucins as biomarkers in glioma but need to be rigorously carried out to distinguish the source of potential promising mucin signals as attributable to tumor vs. normal physiology and tumor progression vs. treatment effects. Ongoing data acquisition, analysis, and sharing are critical to advancing future clinical applications and tackling this complex disease.

## Figures and Tables

**Table 1:** Mucins as candidate precision biomarkers based on literature findings in glioma. Reviews are indicated with **.

Title	Year	Study Type	Disease Entity	Association	Sample Type
Profiling the molecular and clinical landscape of glioblastoma utilizing the oncology research information exchange network brain cancer database (MUC17) [57]	2024	Primary	GBM	Prognosis	Tissue
MUC1 promotes glioblastoma progression and TMZ resistance by stabilizing EGFRVIII [58]	2023	Primary	GBM	Resistance	Cell lines
MUC16 mutation is associated with tumor grade, clinical features, and prognosis in glioma patients [24]	2023	Primary	Glioma	Prognosis	Tissue
MUC17 mutations and methylation are associated with poor prognosis in adult-type diffuse glioma patients [55]	2023	Primary	Diffuse Glioma	Prognosis	Tissue
Mucins as Potential Biomarkers for Early Detection of Cancer [13]	2023	Review **	Various	Detection	Various
The diagnostic and prognostic potential of the EGFR/MUC4/MMP9 axis in glioma patients [49]	2022	Primary	Glioma	Diagnosis/Prognosis	Tissue
MUC21 induces the viability and migration of glioblastoma via the STAT3/AKT pathway [50]	2022	Primary	GBM	Progression	Tissue
Integrative Analysis of MUC4 to Prognosis and Immune Infiltration in Pan-Cancer: Friend or Foe? [52]	2021	Primary	Various	Expression/Prognosis	Tissue
Inhibition of MUC1 exerts cell-cycle arrest and telomerase suppression in glioblastoma cells [59]	2020	Primary	GBM	Resistance	Tissue
MUC15 promotes growth and invasion of glioma cells by activating Raf/MEK/ERK pathway [60]	2020	Primary	Glioma	Progression	Cell Lines
CD146 is highly expressed in glioma stem cells and acts as a cell cycle regulator (MUC18) [61]	2019	Primary	Glioma	Resistance	Cell Lines
Comparative proteogenomic characterization of glioblastoma (MUC19) [62]	2019	Primary	Various	Expression	Tissue
Overexpression of syndecan-1, MUC-1, and putative stem cell markers in breast cancer leptomeningeal metastasis: a cerebrospinal fluid flow cytometry study [63]	2017	Primary	Breast	CSF Expression	CSF
MUC4 modulates human glioblastoma cell proliferation and invasion by upregulating EGFR expression [51]	2014	Primary	GBM	Proliferation/Invasion	Cell Lines/Tissues
Detecting, visualising, and quantifying mucins [64]	2012	Review **	Various	Detection	Various
MUC1 and MUC4: Switching the Emphasis from Large to Small [65]	2011	Review **	Various	Prognosis	Various

**Table 2:** Clinical case studies effectively illustrating the potential and applicability of biospecimens (tissue, blood, CSF) for mucin analysis with clinical implications.

Setting	Tissue	Blood	CSF	Results	Rationale for Clinical Implication
Case study 1: ovarian cancer	Increased expression levels of MUC13 in the tissue of ovarian cancer using immune-histochemistry [85]	Overexpression of MUC16 (CA-125) for detection of ovarian cancer [82]	Serum analysis is more common, but multiple marker analysis is more effective in early diagnosis and could implement CSF in addition to serum [86]	MUC16 is shed from the cell surfaces and enters circulation, leading to elevated levels of MUC16 in serum that are used for ovarian cancer detection	Non-invasive testing like blood sampling can be done more frequently to get a more specific prognosis
Case study 2: breast cancer	High levels of MUC1 were detected in tissue specimens of breast cancer patients [87]	MUC1 was detected in 86% of breast cancer specimens with at least one monoclonal antibody, with elevated levels in IgG and IgM [87]	MUC-1 overexpression was documented on all breast cancer CSF samples analyzed [88]	MUC1 is a promising biomarker and can be measured in tissue, blood, and CSF for the detection and prognosis of breast cancer	More specific information on tumor location could assist in resection and disease monitoring
Case study 3: pancreatic cancer	MUC17 is overexpressed in pancreatic cancer cells when compared with both the normal pancreas and pancreatitis tissues [89]	MUC4 can be used in the diagnosis and prognosis of pancreatic cancer using a SERS-based immunoassay [90]	MUC5AC has been extensively investigated in tissue and sera obtained from pancreatic cancer patients [13]	Mucins are best detected in the serum and tissue of patients with pancreatic cancer. Further analysis of mucins in CSF needs to be done for pancreatic patients	SERS immunoassays can be readily adapted to detect other cancer markers

**Table 3:** Approach, methods, and techniques/analytical tools for each step in the proposed framework for mucin biomarker analysis.

Validation Aspect	Biomarkers	Goal	Approach	Data Type	Potential Methods	Clinical Validation
Discovery	Large sample sets tissue, serum, plasma analyzed (Proteins, metabolites or RNA)	Identify biomarkers	Omics and identify candidates (Measure levels)	Proteomics Metabolomics Levels measured Promising candidates	Gene Set Enrichment Analysis (GSEA), Ingenuity Pathways Analysis (IPA)	Blood Biomarkers in Glioma [107] and Visual Study of Molecular Genotype in Glioma Evolution [108]
Biological	Link to disease process, physiology, biological pathways	Relevance to the disease where employed	Pathophysiological relevance Correlation with clinical parameters Functional relevance	Progression Survival Biol process (e.g., Pathways)	STRING, Reactome, iPathway, PathVisio	Glioblastoma Microenvironment: An Exploratory Study [109]
Clinical	Small cohort to verify candidate markers correlating with disease stage, progression	Test in clinical settings	Disease vs healthy or vs other disease Clinical outcome Stats	Levels across disease stage PPV/NPV, ROC	ClinicalTrials.gov accessed 1 December 2024 [110]	Bevacizumab and Temozolomide Following Radiation and Chemotherapy for Newly Diagnosed Glioblastoma Multiforme [111]
Analytical	Small or large cohort to verify candidate markers correlating with disease stage, progression	Reliable and consistent	Sensitivity/specificity Level of detection Repeated test by other lab or same test, same sample type in independent cohort	How well is it Detected Inter/intra assay Limit of detection Comparison with healthy individuals	Biomarker Comparison Risk Stratification Advanced Analysis Mean Risk Stratification Means to Risk [112]	Temozolomide 12 Cycles Versus 6 Cycles of Standard First-line Treatment in Patients With Glioblastoma [113]

## Data Availability

Not applicable.

## Supplementary Materials

The following supporting information can be downloaded at: https://www.mdpi.com/article/10.3390/biomedicines12122806/s1, Table S1: Analysis of Mucins in Scientific Literature: Publication Trends, Citation Metrics, and Research Impact (1900–2024). Table S2: Protein-Protein Interaction Network Analysis of Mucins, IDH1, MGMT, and EGFR in Glioma: Insights into Molecular Interactions and Pathway Enrichment.

## Abbreviations

GBM	Glioblastoma
MUC	Mucin
PTS	Proline-Threonine-Serine
VWD	Von Willebrand Domain
EGF	Epidermal growth factor
PIK/AKT	Phosphoinositide kinase (PIK)/Protein kinase B
MAPK/ERK	Mitogen-activated protein kinase/Extracellular signal-regulated kinase
Raf	Rapidly accelerated fibrosarcoma
ESCC	Esophageal squamous cell carcinoma
OVGP1	Oviductal glycoprotein one
LGG	Low-grade glioma
CTC	Circulating tumor cell
ctDNA	Circulating tumor DNA
UV	Ultraviolet
CSF	Cerebrospinal fluid

## References

[1] Ostrom Q.T., Price M., Neff C., Cioffi G., Waite K.A., Kruchko C., Barnholtz-Sloan J.S. (2022). CBTRUS Statistical Report: Primary Brain and Other Central Nervous System Tumors Diagnosed in the United States in 2015–2019. Neuro-Oncol..

[2] Berger T.R., Wen P.Y., Lang-Orsini M., Chukwueke U.N. (2022). World Health Organization 2021 Classification of Central Nervous System Tumors and Implications for Therapy for Adult-Type Gliomas: A Review. JAMA Oncol..

[3] Byun Y.H., Park C.K. (2022). Classification and Diagnosis of Adult Glioma: A Scoping Review. Brain Neurorehabil..

[4] Urbańska K., Sokołowska J., Szmidt M., Sysa P. (2014). Glioblastoma multiforme-an overview. Contemp. Oncol..

[5] Stupp R., Mason W.P., Van Den Bent M.J., Weller M., Fisher B., Taphoorn M.J.B., Belanger K., Brandes A.A., Marosi C., Bogdahn U. (2005). Radiotherapy plus Concomitant and Adjuvant Temozolomide for Glioblastoma. N. Engl. J. Med..

[6] Ostrom Q.T., Gittleman H., Xu J., Kromer C., Wolinsky Y., Kruchko C., Barnholtz-Sloan J.S. (2016). CBTRUS Statistical Report: Primary Brain and Other Central Nervous System Tumors Diagnosed in the United States in 2009–2013. Neuro-Oncol..

[7] Ferrer V.P., Moura Neto V., Mentlein R. (2018). Glioma infiltration and extracellular matrix: Key players and modulators. Glia.

[8] Mohiuddin E., Waikimoto H. (2021). Extracellular matrix in glioblastoma: Opportunities for emerging therapeutic approaches. Am. J. Cancer Res..

[9] Obrador E., Moreno-Murciano P., Oriol-Caballo M., López-Blanch R., Pineda B., Gutiérrez-Arroyo J.L., Loras A., Gonzalez-Bonet L.G., Martinez-Cadenas C., Estrela J.M. (2024). Glioblastoma Therapy: Past, Present and Future. Int. J. Mol. Sci..

[10] Karami Fath M., Babakhaniyan K., Anjomrooz M., Jalalifar M., Alizadeh S.D., Pourghasem Z., Abbasi Oshagh P., Azargoonjahromi A., Almasi F., Manzoor H.Z. (2022). Recent Advances in Glioma Cancer Treatment: Conventional and Epigenetic Realms. Vaccines.

[11] Nicholson J.G., Fine H.A. (2021). Diffuse glioma heterogeneity and its therapeutic implications. Cancer Discov..

[12] Dagogo-Jack I., Shaw A.T. (2018). Tumour heterogeneity and resistance to cancer therapies. Nat. Rev. Clin. Oncol..

[13] Gautam S.K., Khan P., Natarajan G., Atri P., Aithal A., Ganti A.K., Batra S.K., Nasser M.W., Jain M. (2023). Mucins as Potential Biomarkers for Early Detection of Cancer. Cancers.

[14] Rachagani S., Torres M.P., Moniaux N., Batra S.K. (2009). Current status of mucins in the diagnosis and therapy of cancer. Biofactors.

[15] Rakha E.A., Boyce R.W.G., Abd El-Rehim D., Kurien T., Green A.R., Paish E.C., Robertson J.F.R., Ellis I.O. (2005). Expression of mucins (MUC1, MUC2, MUC3, MUC4, MUC5AC and MUC6) and their prognostic significance in human breast cancer. Mod. Pathol..

[16] Nagata K., Horinouchi M., Saitou M., Higashi M., Nomoto M., Goto M., Yonezawa S. (2007). Mucin expression profile in pancreatic cancer and the precursor lesions. J. Hepatobiliary Pancreat. Surg..

[17] Boonla C., Wongkham S., Sheehan J.K., Wongkham C., Bhudhisawasdi V., Tepsiri N., Pairojkul C. (2003). Prognostic value of serum MUC5AC mucin in patients with cholangiocarcinoma. Cancer.

[18] Wongkham S., Sheehan J.K., Boonla C., Patrakitkomjorn S., Howard M., Kirkham S., Sripa B., Wongkham C., Bhudhisawasdi V. (2003). Serum MUC5AC mucin as a potential marker for cholangiocarcinoma. Cancer Lett..

[19] Hollingsworth M.A., Swanson B.J. (2004). Mucins in cancer: Protection and control of the cell surface. Nat. Rev. Cancer.

[20] Devine P.L., McKenzie I.F.C. (1992). Mucins: Structure, function, and associations with malignancy. BioEssays.

[21] Gendler S.J., Lancaster C.A., Taylor-Papadimitrou J., Duhig T., Peat N., Burchell J., Pemberton L., Lalani E.N., Wilson D. (1990). Molecular cloning and expression of human tumor-associated polymorphic epithelial mucin. J. Biol. Chem..

[22] Martin Pinzon S., Seeberger P., Silva Varon D. (2019). Mucins and Pathogenic Mucin-Like Molecules Are Immunomodulators During Infection and Targets for Diagnostics and Vaccines. Front. Chem..

[23] Moniaux N. (2001). Structural organization and classification of the human mucin genes. Front. Biosci..

[24] Ferrer V.P. (2023). MUC16 mutation is associated with tumor grade, clinical features, and prognosis in glioma patients. Cancer Genet..

[25] Boltin D., Perets T.T., Vilkin A., Niv Y. (2013). Mucin function in inflamatory bowl disease: An update. J. Clin. Gastroenterol..

[26] Gum J.R., Hicks J.W., Toribara N.W., Siddiki B., Kim Y.S. (1994). Molecular cloning of human intestinal mucin (MUC2) cDNA. Identification of the amino terminus and overall sequence similarity to prepro-von Willebrand factor. J. Biol. Chem..

[27] Timpte C.S., Eckhardt A.E., Abernethy J.L., Hill R.L. (1988). Porcine submaxillary gland apomucin contains tandemly repeated, identical sequences of 81 residues. J. Biol. Chem..

[28] Desseyn J.-L., Aubert J.-P., Porchet N., Laine A. (2000). Evolution of the Large Secreted Gel-Forming Mucins. Mol. Biol. Evol..

[29] Pigny P., Guyonnet-Duperat V., Hill A.S., Pratt W.S., Galiegue-Zouitina S., d’Hooge M.C., Laine A., Van-Seuningen I., Degand P., Gum J.R. (1996). Human mucin genes assigned to 11p15.5: Identification and organization of a cluster of genes. Genomics.

[30] Martínez-Sáez N., Peregrina J.M., Corzana F. (2017). Principles of mucin structure: Implications for the rational design of cancer vaccines derived from MUC1-glycopeptides. Chem. Soc. Rev..

[31] Bengal10 Mucin Structure. https://commons.wikimedia.org/wiki/File:Mucin_Structure.svg.

[32] BruceBlaus Respiratory Epithelium. https://commons.wikimedia.org/wiki/File:Blausen_0766_RespiratoryEpithelium-es.png.

[33] Rosen Y. Metastatic Ovarian Adenocarcinoma-Pleural Fluid Cell Block CA125 Case 168. https://commons.wikimedia.org/wiki/File:Metastatic_ovarian_adenocarcnioma-_Pleural_fluid_cell_block_CA125_Case_168_(5493910931).jpg.

[34] Suzuki T., Conant A., Curow C., Alexander A., Loffe Y., Unternaehrer J.J. Types of Epithelial-Mesenchymal Transition. https://commons.wikimedia.org/wiki/File:Types_of_epithelial-mesenchymal_transition.jpg.

[35] Zina Deretsky N.S.F. Ulcer-Causing Bacterium (*H. pylori*) Crossing Mucus Layer of Stomach. https://commons.wikimedia.org/wiki/File:Ulcer-causing_Bacterium_(H.Pylori)_Crossing_Mucus_Layer_of_Stomach_(4822021538).jpg.

[36] Carraway K.L., Ramsauer V.P., Haq B., Carothers Carraway C.A. (2003). Cell signaling through membrane mucins. BioEssays.

[37] Gum J.R., Ho J.J.L., Pratt W.S., Hicks J.W., Hill A.S., Vinall L.E., Roberton A.M., Swallow D.M., Kim Y.S. (1997). MUC3 Human Intestinal Mucin. J. Biol. Chem..

[38] Bose M., Sanders A., Handa A., Vora A., Cardona M.R., Brouwer C., Mukherjee P. (2024). Molecular crosstalk between MUC1 and STAT3 influences the anti-proliferative effect of Napabucasin in epithelial cancers. Sci. Rep..

[39] Carraway K.L. (2000). Multiple facets of sialomucin complex/MUC4, a membrane mucin and ErbB2 ligand, in tumors and tissues (Y2K update). Front. Biosci..

[40] Karar J., Maity A. (2011). PI3K/AKT/mTOR Pathway in Angiogenesis. Front. Mol. Neurosci..

[41] Jepson S., Komatsu M., Haq B., Arango M.E., Huang D., Carraway C.A.C., Carraway K.L. (2002). Muc4/sialomucin complex, the intramembrane ErbB2 ligand, induces specific phosphorylation of ErbB2 and enhances expression of p27kip, but does not activate mitogen-activated kinase or protein kinaseB/Akt pathways. Oncogene.

[42] Av K. Web of Science Search. https://www.webofscience.com/.

[43] Jonckheere N., Skrypek N., Van Seuningen I. (2014). Mucins and tumor resistance to chemotherapeutic drugs. Biochim. Biophys. Acta.

[44] Theodoropoulos G., Carraway K.L. (2007). Molecular signaling in the regulation of mucins. J. Cell Biochem..

[45] Brown D.V., Daniel P.M., D’Abaco G.M., Gogos A., Ng W., Morokoff A.P., Mantamadiotis T. (2015). Coexpression analysis of CD133 and CD44 identifies proneural and mesenchymal subtypes of glioblastoma multiforme. Oncotarget.

[46] Lin H., Liu C., Hu A., Zhang D., Yang H., Mao Y. (2024). Understanding the immunosuppressive microenvironment of glioma: Mechanistic insights and clinical perspectives. J. Hematol. Oncol..

[47] Wang Q., Li P., Li A., Jiang W., Wang H., Wang J., Xie K. (2012). Plasma specific miRNAs as predictive biomarkers for diagnosis and prognosis of glioma. J. Exp. Clin. Cancer Res..

[48] Machado G.C., Ferrer V.P. (2024). *MUC1* and *MUC4* expression are inversely correlated and trigger immunological response and transport pathways in gliomas. medRxiv.

[49] Quesnel A., Coles N., Polvikoski T.M., Karagiannis G.S., Angione C., Islam M., Khundakar A.A., Filippou P.S. (2022). The diagnostic and prognostic potential of the EGFR/MUC4/MMP9 axis in glioma patients. Sci. Rep..

[50] Wang L., Zhang X., Liu J., Liu Q. (2022). MUC21 induces the viability and migration of glioblastoma via the STAT3/AKT pathway. Exp. Ther. Med..

[51] Li W., Wu C., Yao Y., Dong B., Wei Z., Lv X., Zhang J., Xu Y. (2014). MUC4 modulates human glioblastoma cell proliferation and invasion by upregulating EGFR expression. Neurosci. Lett..

[52] Gao X.P., Dong J.J., Xie T., Guan X. (2021). Integrative Analysis of MUC4 to Prognosis and Immune Infiltration in Pan-Cancer: Friend or Foe?. Front. Cell Dev. Biol..

[53] Huang K., Liu X., Li Y., Wang Q., Zhou J., Wang Y., Dong F., Yang C., Sun Z., Fang C. (2019). Genome-Wide CRISPR-Cas9 Screening Identifies NF-κB/E2F6 Responsible for EGFRvIII-Associated Temozolomide Resistance in Glioblastoma. Adv. Sci..

[54] Nath S., Mukherjee P. (2014). MUC1: A multifaceted oncoprotein with a key role in cancer progression. Trends Mol. Med..

[55] Machado G.C., Ferrer V.P. (2023). MUC17 mutations and methylation are associated with poor prognosis in adult-type diffuse glioma patients. J. Neurol. Sci..

[56] Bhatia R., Gautam S.K., Cannon A., Thompson C., Hall B.R., Aithal A., Banerjee K., Jain M., Solheim J.C., Kumar S. (2019). Cancer-associated mucins: Role in immune modulation and metastasis. Cancer Metastasis Rev..

[57] Demetriou A.N., Chow F., Craig D.W., Webb M.G., Ormond D.R., Battiste J., Chakravarti A., Colman H., Villano J.L., Schneider B.P. (2024). Profiling the molecular and clinical landscape of glioblastoma utilizing the Oncology Research Information Exchange Network brain cancer database. Neurooncol Adv..

[58] Tong F., Zhao J.X., Fang Z.Y., Cui X.T., Su D.Y., Liu X., Zhou J.H., Wang G.X., Qiu Z.J., Liu S.Z. (2023). MUC1 promotes glioblastoma progression and TMZ resistance by stabilizing EGFRvIII. Pharmacol. Res..

[59] Kim S., Seo Y., Chowdhury T., Yu H.J., Lee C.E., Kim K.-M., Kang H., Kim H.J., Park S.-J., Kim K. (2020). Inhibition of MUC1 exerts cell-cycle arrest and telomerase suppression in glioblastoma cells. Sci. Rep..

[60] Cheng M., Liu L. (2020). MUC15 promotes growth and invasion of glioma cells by activating Raf/MEK/ERK pathway. Clin. Exp. Pharmacol. Physiol..

[61] Yawata T., Higashi Y., Kawanishi Y., Nakajo T., Fukui N., Fukuda H., Ueba T. (2019). CD146 is highly expressed in glioma stem cells and acts as a cell cycle regulator. J. Neurooncol.

[62] Asif S., Fatima R., Krc R., Bennett J., Raza S. (2019). Comparative proteogenomic characterization of glioblastoma. CNS Oncol..

[63] Cordone I., Masi S., Summa V., Carosi M., Vidiri A., Fabi A., Pasquale A., Conti L., Rosito I., Carapella C.M. (2017). Overexpression of syndecan-1, MUC-1, and putative stem cell markers in breast cancer leptomeningeal metastasis: A cerebrospinal fluid flow cytometry study. Breast Cancer Res..

[64] Harrop C.A., Thornton D.J., McGuckin M.A. (2012). Detecting, Visualising, and Quantifying Mucins.

[65] Albrecht H., Carraway K.L. (2011). MUC1 and MUC4: Switching the emphasis from large to small. Cancer Biother. Radiopharm..

[66] Zhang S., Zhang W., Xiao Y., Qin T., Yue Y., Qian W., Shen X., Ma Q., Wang Z. (2020). Targeting MUC15 Protein in Cancer: Molecular Mechanisms and Therapeutic Perspectives. Curr. Cancer Drug Targets.

[67] Osti D., Del Bene M., Rappa G., Santos M., Matafora V., Richichi C., Faletti S., Beznoussenko G.V., Mironov A., Bachi A. (2019). Clinical Significance of Extracellular Vesicles in Plasma from Glioblastoma Patients. Clin. Cancer Res..

[68] Heimberger A.B., McGary E.C., Suki D., Ruiz M., Wang H., Fuller G.N., Bar-Eli M. (2005). Loss of the AP-2alpha transcription factor is associated with the grade of human gliomas. Clin. Cancer Res..

[69] Yi Y., Kamata-Sakurai M., Denda-Nagai K., Itoh T., Okada K., Ishii-Schrade K., Iguchi A., Sugiura D., Irimura T. (2010). Mucin 21/epiglycanin modulates cell adhesion. J. Biol. Chem..

[70] GongSun X., Zhao Y., Jiang B., Xin Z., Shi M., Song L., Qin Q., Wang Q., Liu X. (2019). Inhibition of MUC1-C regulates metabolism by AKT pathway in esophageal squamous cell carcinoma. J. Cell Physiol..

[71] Pino V., Ramsauer V.P., Salas P., Carraway C.A.C., Carraway K.L. (2006). Membrane Mucin Muc4 Induces Density-dependent Changes in ERK Activation in Mammary Epithelial and Tumor Cells. J. Biol. Chem..

[72] von Mering C., Huynen M., Jaeggi D., Schmidt S., Bork P., Snel B. (2003). STRING: A database of predicted functional associations between proteins. Nucleic Acids Res..

[73] Lam W.K.J., Chan K.C.A. (2019). Plasma DNA for early cancer detection-opportunities and challenges. Expert. Rev. Mol. Diagn..

[74] Tang Q., Cheng J., Cao X., Surowy H., Burwinkel B. (2016). Blood-based DNA methylation as biomarker for breast cancer: A systematic review. Clin. Epigenet..

[75] Giuntoli R.L., Rodriguez G.C., Whitaker R.S., Dodge R., Voynow J.A. (1998). Mucin gene expression in ovarian cancers. Cancer Res..

[76] Jonckheere N., Vincent A., Neve B., Van Seuningen I. (2021). Mucin expression, epigenetic regulation and patient survival: A toolkit of prognostic biomarkers in epithelial cancers. Biochim. Biophys. Acta Rev. Cancer.

[77] Lakshmanan I., Ponnusamy M.P., Macha M.A., Haridas D., Majhi P.D., Kaur S., Jain M., Batra S.K., Ganti A.K. (2015). Mucins in lung cancer: Diagnostic, prognostic, and therapeutic implications. J. Thorac. Oncol..

[78] Byrd J.C., Bresalier R.S. (2004). Mucins and mucin binding proteins in colorectal cancer. Cancer Metastasis Rev..

[79] Krishn S.R., Kaur S., Smith L.M., Johansson S.L., Jain M., Patel A., Gautam S.K., Hollingsworth M.A., Mandel U., Clausen H. (2016). Mucins and associated glycan signatures in colon adenoma-carcinoma sequence: Prospective pathological implication(s) for early diagnosis of colon cancer. Cancer Lett..

[80] Yan P.S., Ho S.B., Itzkowitz S.H., Byrd J.C., Siddiqui B., Kim Y.S. (1990). Expression of native and deglycosylated colon cancer mucin antigens in normal and malignant epithelial tissues. Lab. Investig..

[81] Mikkelsen V.E., Solheim O., Salvesen Ø., Torp S.H. (2021). The histological representativeness of glioblastoma tissue samples. Acta Neurochir..

[82] Aithal A., Rauth S., Kshirsagar P., Shah A., Lakshmanan I., Junker W.M., Jain M., Ponnusamy M.P., Batra S.K. (2018). MUC16 as a novel target for cancer therapy. Expert. Opin. Ther. Targets.

[83] Atlas H.P. Cell Type Markers. The Human Protein Atlas.

[84] Boland J.L., Zhou Q., Iasonos A.E., O’Cearbhaill R.E., Konner J., Callahan M., Friedman C., Aghajanian C., Sabbatini P., Zamarin D. (2020). Utility of serum CA-125 monitoring in patients with ovarian cancer undergoing immune checkpoint inhibitor therapy. Gynecol. Oncol..

[85] Chauhan S.C., Kumar D., Jaggi M. (2009). Mucins in ovarian cancer diagnosis and therapy. J. Ovarian Res..

[86] Zhang Z., Yu Y., Xu F., Berchuck A., van Haaften-Day C., Havrilesky L.J., de Bruijn H.W., van der Zee A.G., Woolas R.P., Jacobs I.J. (2007). Combining multiple serum tumor markers improves detection of stage I epithelial ovarian cancer. Gynecol. Oncol..

[87] Croce M.V., Isla-Larrain M.T., Demichelis S.O., Gori J.R., Price M.R., Segal-Eiras A. (2003). Tissue and serum MUC1 mucin detection in breast cancer patients. Breast Cancer Res. Treat..

[88] Simon M.J., Iliff J.J. (2016). Regulation of cerebrospinal fluid (CSF) flow in neurodegenerative, neurovascular and neuroinflammatory disease. Biochim. Biophys. Acta.

[89] Moniaux N., Andrianifahanana M., Brand R.E., Batra S.K. (2004). Multiple roles of mucins in pancreatic cancer, a lethal and challenging malignancy. Br. J. Cancer.

[90] Wang G., Lipert R.J., Jain M., Kaur S., Chakraboty S., Torres M.P., Batra S.K., Brand R.E., Porter M.D. (2011). Detection of the potential pancreatic cancer marker MUC4 in serum using surface-enhanced Raman scattering. Anal. Chem..

[91] Ahmed R., Oborski M.J., Hwang M., Lieberman F.S., Mountz J.M. (2014). Malignant gliomas: Current perspectives in diagnosis, treatment, and early response assessment using advanced quantitative imaging methods. Cancer Manag. Res..

[92] Wirsching H.G., Galanis E., Weller M. (2016). Glioblastoma. Handb. Clin. Neurol..

[93] Shankar G.M., Balaj L., Stott S.L., Nahed B., Carter B.S. (2017). Liquid biopsy for brain tumors. Expert. Rev. Mol. Diagn..

[94] Idrees B.S., Teng G., Israr A., Zaib H., Jamil Y., Bilal M., Bashir S., Khan M.N., Wang Q. (2023). Comparison of whole blood and serum samples of breast cancer based on laser-induced breakdown spectroscopy with machine learning. Biomed. Opt. Express.

[95] Kawa I.A., Masood A., Amin S., Mustafa M.F., Rashid F. (2019). Clinical Perspective of Posttranslational Modifications.

[96] Litvinov S.V., Hilkens J. (1993). The epithelial sialomucin, episialin, is sialylated during recycling. J. Biol. Chem..

[97] Thingstad T., Vos H.L., Hilkens J. (2001). Biosynthesis and shedding of epiglycanin: A mucin-type glycoprotein of the mouse TA3Ha mammary carcinoma cell. Biochem. J..

[98] Linden S.K., Sutton P., Karlsson N.G., Korolik V., McGuckin M.A. (2008). Mucins in the mucosal barrier to infection. Mucosal Immunol..

[99] Erickson A., Krauze A.V. Proposed Framework for Mucin Analysis as Precision Biomarkers in Glioma Illustrating Potential Steps That May Be Carried out Concurrently or Sequentially. https://BioRender.com.

[100] Jung M., Klotzek S., Lewandowski M., Fleischhacker M., Jung K. (2003). Changes in concentration of DNA in serum and plasma during storage of blood samples. Clin. Chem..

[101] Cordova C., Syeda M.M., Corless B., Wiggins J.M., Patel A., Kurz S.C., Delara M., Sawaged Z., Utate M., Placantonakis D. (2019). Plasma cell-free circulating tumor DNA (ctDNA) detection in longitudinally followed glioblastoma patients using *TERT* promoter mutation-specific droplet digital PCR assays. J. Clin. Oncol..

[102] Au K.L.K., Latonas S., Shameli A., Auer I., Hahn C. (2020). Cerebrospinal Fluid Flow Cytometry: Utility in Central Nervous System Lymphoma Diagnosis. Can. J. Neurol. Sci..

[103] Akers J.C., Hua W., Li H., Ramakrishnan V., Yang Z., Quan K., Zhu W., Li J., Figueroa J., Hirshman B.R. (2017). A cerebrospinal fluid microRNA signature as biomarker for glioblastoma. Oncotarget.

[104] Zubair A., Orlando D.J. (2023). Ommaya Reservoir.

[105] Rickert C.A., Lutz T.M., Marczynski M., Lieleg O. (2020). Several Sterilization Strategies Maintain the Functionality of Mucin Glycoproteins. Macromol. Biosci..

[106] Debailleul V., Laine A., Huet G., Mathon P., d’Hooghe M.C., Aubert J.P., Porchet N. (1998). Human mucin genes MUC2, MUC3, MUC4, MUC5AC, MUC5B, and MUC6 express stable and extremely large mRNAs and exhibit a variable length polymorphism. An improved method to analyze large mRNAs. J. Biol. Chem..

[107] Blood Biomarker Signature in Glioma. https://clinicaltrials.gov/study/NCT03698201?cond=Glioma%20of%20Brain&term=biomarker&rank=1.

[108] Visual Study of Molecular Genotype in Glioma. https://clinicaltrials.gov/study/NCT03750890?cond=Glioma%20of%20Brain&term=biomarker&rank=2.

[109] Glioma Microenvironment an Exploratory Study. https://clinicaltrials.gov/study/NCT03189420?cond=Glioma%20of%20Brain&rank=5.

[110] Clinical Trials. https://clinicaltrials.gov/.

[111] Bevacizumab and Temozolomide Following Radiation and Chemotherapy for Newly Diagnosed Glioblastoma Multiforme. https://clinicaltrials.gov/study/NCT00590681?cond=Glioblastoma&aggFilters=results:with&page=2&rank=20.

[112] Biomarker Tools. https://analysistools.cancer.gov/biomarkerTools/.

[113] Temozolomide 12 Cycles Versus 6 Cycles of Standard First-Line Treatment in Patients with Glioblastoma. https://clinicaltrials.gov/study/NCT02209948?cond=Glioblastoma&term=biomarker&aggFilters=results:with&page=2&rank=13.

[114] Ganguly K., Rauth S., Marimuthu S., Kumar S., Batra S.K. (2020). Unraveling mucin domains in cancer and metastasis: When protectors become predators. Cancer Metastasis Rev..

[115] Goyette M.A., Stevens L.E., DePinho C.R., Seehawer M., Nishida J., Li Z., Wilde C.M., Li R., Qiu X., Pyke A.L. (2024). Cancer-stromal cell interactions in breast cancer brain metastases induce glycocalyx-mediated resistance to HER2-targeting therapies. Proc. Natl. Acad. Sci. USA.

[116] Chaudhary S., Siddiqui J.A., Appadurai M.I., Maurya S.K., Murakonda S.P., Blowers E., Swanson B.J., Nasser M.W., Batra S.K., Lakshmanan I. (2024). Dissecting the MUC5AC/ANXA2 signaling axis: Implications for brain metastasis in lung adenocarcinoma. Exp. Mol. Med..

[117] Detappe A., Mathieu C., Jin C., Agius M.P., Diringer M.C., Tran V.L., Pivot X., Lux F., Tillement O., Kufe D. (2020). Anti-MUC1-C Antibody-Conjugated Nanoparticles Potentiate the Efficacy of Fractionated Radiation Therapy. Int. J. Radiat. Oncol. Biol. Phys..

[118] Maji S., Chaudhary P., Akopova I., Nguyen P.M., Hare R.J., Gryczynski I., Vishwanatha J.K. (2017). Exosomal Annexin II Promotes Angiogenesis and Breast Cancer Metastasis. Mol. Cancer Res..

[119] Coletto E., Savva G.M., Latousakis D., Pontifex M., Crost E.H., Vaux L., Telatin A., Bergstrom K., Vauzour D., Juge N. (2023). Role of mucin glycosylation in the gut microbiota-brain axis of core 3 O-glycan deficient mice. Sci. Rep..

